# Definition of Microscopic Tumor Clearance (R0) in Pancreatic Cancer Resections

**DOI:** 10.3390/cancers2042001

**Published:** 2010-11-25

**Authors:** Anna Melissa Schlitter, Irene Esposito

**Affiliations:** 1 Institute of Pathology, Technische Universität München, Ismaningerstr. 22, 81675 Munich Germany; E-Mail: melissa.schlitter@lrz.tum.de; 2 Institute of Pathology, Helmholtz Zentrum München, Ingolstädter Landstraße 1, 85764 Neuherberg, Germany

**Keywords:** pancreatic cancer, resection margin, R1, definition of resection status, pathological standardization

## Abstract

To date, curative resection is the only chance for cure for patients suffering from pancreatic ductal adenoacarcinoma (PDAC). Despite low reported rates of microscopic tumor infiltration (R1) in most studies, tumor recurrence is a common finding in patients with PDAC and contributes to extremely low long-term survival rates. Lack of international definition of resection margins and of standardized protocols for pathological examination lead to high variation in reported R1 rates. Here we review recent studies supporting the hypothesis that R1 rates are highly underestimated in certain studies and that a microscopic tumor clearance of at least 1 mm is required to confirm radicality and to serve as a reliable prognostic and predictive factor.

## 1. Introduction

Pancreatic ductal adenoacarcinoma (PDAC) is one of the most aggressive tumors with an extremely poor prognosis. Despite recent advances in surgical treatment and adjuvant therapy, the survival rates are still very low (five-year survival about 5%) [1,2]. To date, curative resection is the only chance for cure and prolonged survival for a minority of patients (10–20%) affected by pancreatic cancer [1,3]. Determination of the resection status is part of the pathological examination and is a crucial step in adequate staging and planning of consecutive treatment. Moreover, it has been shown to be a prognostic factor for PDAC in several studies [4,5,6,7,8]. Still, no consensus exists concerning the exact definition of microscopic tumor clearance (R0) and the standardization of pathological reporting.

The reported microscopic tumor infiltration (R1) rates show a surprisingly high variation ranging from 17% to 85% (Table 1). In divergence with low reported R1 rates, local recurrence is a current problem for PDAC and concerns up to 87% of patients [9,10,11,12]. This obvious discrepancy is well shown by a recent retrospective study including 360 patients with a local recurrence rate of more than 66% of initially R0 diagnosed patients. Interestingly, the initial R1 group (17%) showed a comparable recurrence rate of 68% [9]. This findings support the hypothesis that R1 rates are highly underestimated in certain studies. Divergent definitions of resection margins and lack of a standardized pathological examination protocol are probably the main reason for the high variation in reported R1 rates.

**Table 1:** Comparison of R1 rates for PDAC.

Study	Year	Study period	Number of patients	R1/R2 rates
Willet *et al.* [8]	1993	1978–1991	72	51%
Yeo *et al.* [43]	1997	1990–1996	282	29%
Richter *et al.* [6]	2003	1972–1998	194	37%
Wagner *et al.* [7]	2004	1993–2001	165	23.6%
Cameron *et al.* [44]	2006	1969–2003	405	36%
Kuhlmann *et al.* [45]	2006	1992–2001	160	50%
Verbeke *et al.* [17]	2006	1995–2003	26	85%
Winter *et al.* [46]	2006	1970–2006	1175	42%
Raut *et al.* [9]	2007	1990–2004	360	17%
Esposito *et al.* [16]	2008	2005–2006	111	76%
Campbell *et al.* [18]	2009	1997–2007	163	79%
Jamieson *et al.* [4]	2010	1996–2007	148	74%

## 2. Definition of the Resection Margins

The lack of consensus regarding definition of the relevant margins and the absence of a standardized nomenclature are recognized problems in pathological reporting for pancreatic resections with PDAC [13].

The pancreas is located in the retroperitoneum. Surgical procedures for pancreatic resections include transection and mobilization of retroperitoneal surfaces. Furthermore, PDAC is characterized by an infiltrative growth and invasion of adjacent structures occurring in early stages. Due to its special anatomical position and the characteristic growth pattern, all transection and circumferential margins have to be analyzed in order to evaluate the radicality of the pancreas resection. The relevant margins involve the “true” transection margins and the circumferential resection margins. Thetransection margins of a pancreatoduodenectomy comprise: the pancreatic duct margin (pancreatic neck margin), the bile duct margin, the proximal duodenal/stomach margin and the distal duodenal margin. The circumferential resection margins include: the posterior pancreatic surface, the medial margin (groove along the superior mesenteric vein/portal vein) and the anterior surface. The anterior surface is a particular case since it is not a true surgical margin but a dissection space from the surrounding surfaces. However, a prognostic value of invasion of the anterior surface has been shown [14,15]. In case of a vascular resection, the entire transection margins of the vessel should be examined [16,17].

Little is known about prognostic differences of specific sites of margin infiltration. A recent study examined this aspect, showing that the involvement of the margins requiring lympho-vascular division (medial margin and pancreatic resection margin), in contrast to margins that involve a mobilization phase (including posterior margin, anterior surface and duodenal serosa), is associated with a significantly shorter median survival (11.1 months *versus* 18.9 months) [4].

**Figure 1:**
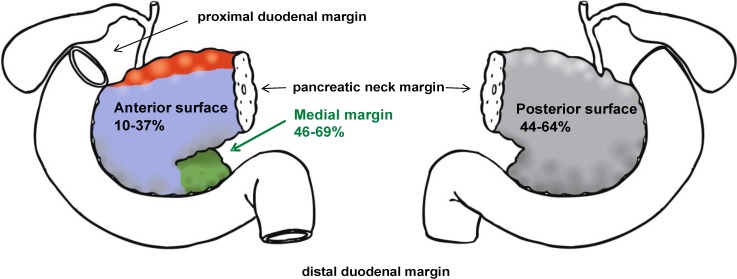
Pancreatoduodenectomy specimens: The posterior surface and the medial margin are the main sites of microscopic tumor infiltration [4,16,17,18]. Drawings by Lukas Bauer.

Systematic investigations of all relevant margins demonstrate that the posterior surface and the medial margin are the main sites of microscopic tumor infiltration: the medial margin is concerned in 46–69% of cases, the posterior surface in 44% to 64%, (Figure 1, Table 2) [4,16,17,18]. The majority of investigated specimens (55–68%) show involvement of a single margin whereas in about one-third of cases two or more margins are involved (Table 2) [4,16,17,18].

**Table 2:** Comparison of four large studies using a standardized protocol.

Parameter	Esposito *et al.* [16]	Verbeke *et al.* [17]	Jamieson *et al.* [4]	Campbell *et al.* [18]
Protocol	RCPath guidelines	RCPath guidelines	RCPath guidelines	RCPath guidelines
Cases	111	26	148	163
Study period	2005-2006	1995-2003	1996-2007	1997-2007
Margin definition	</= 1 mm	</= 1 mm	</= 1 mm	</= 1 mm
**R-classification**				
R0	24%	15%	26%	21%
R1 (1 mm rule)	76%	85%	74%	79%
*R1 (0 mm rule)*	*No data*	*No data*	*55%*	*45%*
**Margin involvement**				
Posterior	47%	64%	44%	54%
Medial	69%	55%	46%	50%
Anterior surface	10%	18%	37%	*
Pancreatic duct	4%	9%		
Bile duct	5%	0	3%	3%
Stomach/Duodenum	4%	0	2%	5%
Transection				30%
Only one single margin involved	68%	55%	58%	65%
Two or more margins involved (multifocal)	32%	45%	42%	35%

* Isolated infiltration of anterior surface was not considered R1

### Standardization of Pathological Investigation

**Figure 2:**
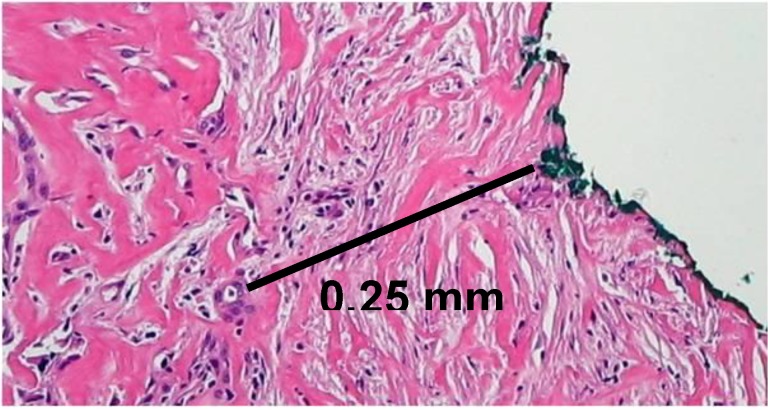
Pancreatoduodenectomy specimen with tumor infiltration (R1): Direct invasion of tumor cells within 1 mm of the medial margin (green).

Standardized pathological reporting taking into consideration all the relevant margins is a further step to achieve meaningful R1 rates. In a recent study, we have shown that the introduction of a standardized protocol for the evaluation of pancreatic resection specimens with PDAC led to a 5.4 fold higher R1 rate compared to the R1 rate recorded in the same institution and with the same operating surgical team without the use of a standardized protocol (76% *versus* 14%) [16]. The observation that a standardized examination influences the reporting of resection status is supported by further studies [17,19]. Liska *et al.* [19] report an increase in R1 rates by stepwise introduction of a detailed standardized protocol starting from an initial rate of 23.5% to 40% and finally to 53.8%. Interestingly, four recent studies from different institutions all based on a similar standardized protocol showed analogous results concerning most relevant pathologic parameters, including the highest reported R1 rates for PDAC to date (74% to 85%, Table 3) [4,16,17,18]. This standardized protocol based on inking of the specimens according to a defined color code and slicing of the specimen perpendicular to the long axis of the duodenum has been described in detail elsewhere (for an example of microscopical determination of resection margin, see Figure 2) [13,16].

Taken together, these data clearly show that the relatively high R1 rate after surgical resection of PDAC does not reflect the surgical quality, as stated by others [9], but is more the result of careful pathological investigation.

## 3. Margin Clearance

As stated above, the determination of the resection status is an essential part of pathological examination. The classifications of the International Union Against Cancer (UICC, www.uicc.org) and the American Joint Committee on Cancer (AJCC, www.cancerstaging.org) make a distinction between negative resection margins (R0), microscopic tumor infiltration (R1) and macroscopic residual tumor (R2). UICC defines R1 as “the presence of residual tumor after treatment” without specific histological definition [20]. Further histological definitions of margin clearance exist on a national level. In North America, guidelines define microscopic residual tumor as the presence of tumor cells at the surface of the resection margin (0 mm rule) [21] whereas guidelines of the British Royal College of Pathology (RCPath) define R1 as the presence of tumor cells within 1 mm of the resection margin (www.rcpath.org; Figure 2). The lack of international consensus for the definition of margin involvement clearly contributes to the high variation in the reported R1 rates. Application of the 1 mm rule as defined by RCPath guidelines reveals a 1.3 to 1.8 fold higher R1 rate when compared to the 0 mm rule according to UICC in PDAC cohorts (Table 3) [4,18,22].

**Table 3:** Comparison of R1 rates for PDAC between application of the UICC and RCPath criteria.

Study	Year	R1 RCPath (1 mm rule)	R1 UICC (0 mm rule)	Ratio RCPath/ UICC
Jamieson *et al.* [4]	2009	74%	55%	1.4
Campbell *et al.* [18]	2009	79%	45%	1.8
Gaedcke *et al.* [22]	2009	82.6% (R1/R2)	63% (R1/R2)	1.3

Evidence that a minimum clearance of more than 1 mm is required to achieve complete surgical resection comes from different recent studies. Applying the resection margin definition of the Royal College of Pathology, Campbell *et al.* [18] classified 79% of investigated resections (128 of 163 cases) as R1. Fifty-five percent of the R1 cases showed a direct involvement (“unequivocal" margin infiltration) of the margin, and in 45% of the R1 cases tumor cells were found within 1 mm of the resection margin (“equivocal” margin infiltration). Retrospectively, these “equivocal” cases had a median survival of 15.4 months, more comparable to the median survival of the “unequivocal” group with 12.6 months than the clearly prolonged median survival of the R0 group (25.4 months). Moreover, equivocal and unequivocal R1 resections showed no significant difference in overall survival [18]. Indirect evidence comes from a North American molecular study monitoring k-ras mutations in tumor free resection margins. Thirty-seven out of 70 patients (53%) diagnosed with curative resection status according to the North American guidelines (0 mm rule) had k-ras mutations at the investigated surgical margins. Furthermore, k-ras mutation-negative and -positive patients showed a significant difference in overall survival (55 *versus* 15 months) [23]. This observation correlates with the high reported recurrence rates despite initial low R1 rates and points to a very aggressive biological behavior of the tumor cells in PDAC. The first systematic study investigating the relationship between distance of cancer cells from the margin and prognosis was published in 2009 [24]. In this large study of 365 patients, optimal long-term survival (five-year survival of 18.5%) was only achieved for a minimal clearance of more than 1.5 mm. Five-year survival of patients with direct involved margins was comparable to long-term survival of patients with close margins between 0 and 1.5 mm (3.9% *versus* 4.6%). Furthermore, the definition of R1 as tumor cells within 1.5 mm from the resection margin was an independent predictor of survival in multivariate analysis. Consequently, the authors of the study pointed to a possible role of adjuvant radiochemotherapy for patients with a margin clearance less than 1.5 mm [24]. An excellent five-year survival of 68% has been previously reported in a large Japanese study for patients with a margin clearance of >5 mm [14].

The definition after curative resection is a common problem in different tumor entities. In rectal cancer, the prognostic value of a minimal margin clearance of >1 mm for the circumferential resection margin (CRM) is widely accepted and reflected by classification as CRM-positive/negative [25]. Similar data are accessible for esophageal cancer [26,27,28].

Pancreatic cancer and cholangiocarcinoma share biological, pathological and prognostic features. Both tumors have a very poor prognosis with a five-year overall-survival rate less than 5% for patients with cholangiocarcinoma [29]. Similar to pancreatic cancer, cholangiocarcinoma is characterized by infiltrative and discontinuous growth and perineural invasion [30,31]. Furthermore, spread along biliary ducts and longitudinal submucosal extension is common [32,33]. Curative resection is the only potential treatment for patients with cholangiocarcinoma [33,34]. Likewise, the resection status is strongly associated with survival [35,36]. Tumor recurrence is common in bile duct carcinoma: Kabayashi *et al*. [37] report a tumor recurrence of 53% for hilar bile duct carcinoma after R0 resection. As in pancreatic cancer, recent data point to the need of an extended tumor free margin to minimize tumor recurrence. Since the majority (60–70%) of cholangiocarcinoma arise at the bifurcation of the hepatic ducts (hilar bile duct carcinoma, Klatskin tumors), most studies focus on hilar bile duct carcinoma. Data from a Japanese study show that a minimal tumor free margin of 5 mm is required for hilar bile duct carcinoma to avoid anastomotic tumor recurrence [32], concordantly to proposal of the Japanese Society of Biliary Surgery of tumor-free margins of 5 mm in duodenal and hepatic direction [38]. A second study has confirmed the data by showing that a margin >5 mm provided a significantly better long-term survival than closer margins. Furthermore, no significant difference was observed between the R1 and R0 group with narrow margins closer than 5 mm [34]. An additional problem concerns the prognostic value of dysplasia at the margin clearance, which is often observed at the margin. Whereas invasive carcinoma at the resection margin is a negative prognostic factor, recent studies have shown that presence of carcinoma *in situ* is not associated with poor prognosis [39,40,41].

## 4. Conclusions and Future Perspectives

Altogether, these data indicate how a definition of the resection margin status that takes into consideration the biology of the single tumor entities can be a reliable prognostic and predictive factor as well as a guide for further treatment options. Concerning pancreatic cancer, further systematic investigations are certainly needed to determine a margin clearance with a high prognostic value, as it has been previously shown in rectal cancer [25,42].

A first step toward standardization could be represented by a modification of the definition of the resection margin status (R factor of the TNM classification of the UICC) that is applicable to all tumor entities but that simultaneously takes into account the biological variability between tumors. A recent publication addressed this topic recognizing the importance of an adjusted R-status definition and proposing an expanded R classification, which includes the statement of the minimal distance between tumor and resection margin for rectal cancer with a possible relevance for other tumor entities [25]. The data discussed in this review strongly support that a meaningful R0 definition for pancreatic cancer requires a minimal clearance of at least 1 mm, and support the implementation of an international expanded R classification as proposed for pancreatic cancer.
